# The random dot tachistogram: a novel task that elucidates the functional architecture of decision

**DOI:** 10.1038/srep30787

**Published:** 2016-07-29

**Authors:** Wilfried Genest, Robert Hammond, R. H. S. Carpenter

**Affiliations:** 1 Department of Physiology, Development and Neuroscience, University of Cambridge, CB2 3EG UK

## Abstract

Reaction times are long and variable, almost certainly because they result from a process that accumulates noisy decision signals over time, rising to a threshold. But the origin of the variability is still disputed: is it because the incoming sensory signals are themselves noisy? Or does it arise within the brain? Here we use a stimulus – the *random dot tachistogram* – which demands spatial integration of information presented essentially instantaneously; with it, we demonstrate three things. First, that the latency distributions still show the variability characteristic of LATER, implying that there must be two integrators in series. Secondly, that since this variability persists despite removal of all temporal noise from the stimulus, or even trial-to-trial spatial variation, it must come from within the nervous system. Finally, that the average rate of rise of the decision signal depends linearly on how many dots move in a given direction. Taken together, this suggests a rather simple, two-stage model of the overall process. The first, detection, stage performs local temporal integration of stimuli; the local, binary, outcomes are linearly summed and integrated by LATER units in the second stage, that perform the final global decision by a process of racing competition.

Two features of reaction times are surprising. One is that they are extraordinarily *long*: even saccades, the rapid eye movements that have evolved to be one of the fastest movements that we make, have a reaction time or latency of the order of 200 ms, much longer than expected from considerations of conduction time, receptor activation and synaptic delay. The other is that they are extraordinarily *variable*: when experimental conditions are kept absolutely constant in a sequence of trials, identical visual stimuli evoke saccades with latencies that can vary randomly from some 100 to 300 ms. It is generally agreed that the length of reaction times reflects the time it takes for a decision signal to be accumulated to threshold, and that the variability reflects the noisiness of this integrated signal. But what is more controversial is the *origin* of this noise. For a long time it was assumed to represent the inevitable noisiness of afferent sensory signals; the possibility that it might also be introduced within the brain itself has often been suggested but never proved incontrovertibly.

One reason for the current lack of agreement on this point is the existence of two strands of experimental approach, using fundamentally different kinds of stimuli, greatly influencing the interpretation of results. Some use stimuli with a great deal of random variability built into them. A popular example is the random dot kinetogram or RDK^1^, in which a subject estimates the overall direction of movement of a set of random dots: as would be expected, the integration of the noise in the stimulus generates variability of response time^2^. Others use stimuli utterly different from the RDK: localized, with high contrast and signal-to-noise ratios, and presented statically, just once^3^,^4^,^5^,^6^. If variability of reaction time were essentially due to sensory noise, with such stimuli we should see scarcely any. Yet a bright target suddenly presented in the periphery typically evokes a saccade with a latency that is *still* extremely variable, implying that much – perhaps most - of this variability is generated by the system itself – the sensory receptors and muscles, but above all, the neural processes within the brain itself.

Here we use a relatively unfamiliar kind of oculomotor task, using what we call a *random dot tachistogram* or RDT (ταχιστος = ‘instantaneous’, as in the familiar tachistoscope). We present subjects with a spatially random set of dots, but instead of the continuous, sustained movement characteristic of the RDK, at time zero the dots undergo a *single* displacement randomly to the left or right, between consecutive frames (Fig. 1). Thus it is truly instantaneous, in that both the initial and the final configuration of dots contain no information relevant to the task, which is generated only at the instant of changeover. Retaining the important feature of the RDK that it is distributed and therefore demands spatial integration, it avoids the potential confound that the RDK necessarily injects continual external noise into the decision system. This type of stimulus seems first to have been introduced by Anstis^7^, as a kind of temporal analogue of the Julesz random-dot stereo patterns, and used extensively thereafter to investigate the relation of the consequent phi-perception to the correspondence problem^8^,^9^,^10^. Here we use it rather differently, to demonstrate three things: that almost all of the variability is indeed internally generated, that there are two distinct stages of accumulation, equivalent to detection and decision, and that spatially distributed information from individual detectors combines linearly to determine the rate of rise of the decision process. As a result we can predict the detailed behaviour of individual subjects with a simple model having unusually few free parameters.

## Results

### Latency distributions

It is convenient to present distributions of RTs using *reciprobit plots*, which show cumulative probability with a probit ordinate as a function of reciprocal latency: data following the LATER decision model will then generate a straight line, because (under high-contrast conditions), 1/(RT) should follow a Gaussian distribution^3^. There is not space here to go into the details of LATER (Linear Approach to Threshold with Ergodic Rate), which has been extensively published elsewhere^3^,^11^,^12^ and is described on a website^13^; in essence, a single LATER unit embodies a decision signal *S* that, in response to a stimulus, rises linearly at a rate *r* from an initial value *S*_0_ until it reaches a criterion level *S*_T_, at which point the response is initiated. On different trials, *r* varies randomly as a Gaussian variate (μ, σ), giving rise to random variation in RT, such that its reciprocal is also Gaussian. LATER can be interpreted as a Bayesian process, in which *S* signifies log probability, *S*_0_ represents log prior probability and μ corresponds to the rate of supply of information^14^,^15^.

Since errors occur in the RDT task (when the subject chooses the direction of the minority of dots) it is helpful to show distributions in the form of ‘incomplete’ reciprobit plots representing, for a given degree of *support* (a concept elucidated in the Methods section, under ‘Protocol’) in a particular direction, the cumulation of RTs of all responses in that direction as a proportion of all responses in either direction, asymptoting at the final response rate for that direction, in this case75% and 25% (Fig. 2B).

Figure 2A shows complete reciprobit plots for all subjects, demonstrating that despite eliminating temporal variability in the stimulus, the overall RT still shows considerable randomness, its distribution still as predicted by LATER. This is true even if the spatial randomness is also removed, by using the same starting pattern on each trial and invariant dot movement (Fig. 3A–C). The distributions are then found to be essentially identical, whether the spatial pattern is constant, or whether it varies randomly from trial to trial. In other words, the normal variability of reaction times does not require a noisy stimulus, whether the noise is temporal or spatial. (At these contrast and luminance levels, temporal noise introduced by low-level aspects of the stimuli such as quantum fluctuations, or by such processes as molecular and synaptic diffusion, are also negligible in comparison to the overall variability of reaction time)^16^. Conversely, a direct comparison of latency distributions using RDT and RDK stimuli (Fig. 3D,E) shows the greater range and shallower slope due to the added variance introduced by the latter (more than three times what is seen with RDT) as well as the increased accuracy of the asymptotic responses (84.5% rather than 69.8%), as would be expected from the extra information that can be integrated over time.

A typical set of incomplete distributions for the RDT task across all degrees of support for one subject is shown at the top of Fig. 4. Although the curves have similar shapes, when support is greater responses are faster, and the asymptote is at a higher final response rate. As can be seen in Fig. 4A–E, all subjects exhibited this pattern, though there are scaling differences between subjects, corresponding to the idiosyncratic variation in the underlying parameters characteristic of latency distributions in general.

Using a model consisting of two racing LATER units (correct and incorrect), with different mean rates of rise, μ, but the same variance, we performed Monte-Carlo iterative simulations as described in Methods, separately for each participant and for each degree of support, to estimate the two values of μ (μ_i_ and μ_c_) that yielded the best fit of the simulated latency distributions to the observed ones: Fig. 4A–E, shows the degree of correspondence between observed and simulated distributions for all subjects for supports of 0%, 25%, 75% and 100%; in no case was *p* < 0.583 (KS 2).

As well as accounting rather precisely for the shapes of the latency distributions of correct and incorrect responses, the model also predicted the final asymptotic response rates almost perfectly across all values of support and all participants (Fig. 5).

### Mean rate of rise, μ

We found that μ increased linearly with increasing stimulus support for that direction (Fig. 6), although the slope varied somewhat between participants (final panel of Fig. 6), and to a much smaller extent, the offset. This means that the number of free parameters required to explain all the data for all degrees of support for any subject is reduced to just two (the slope and offset), with the two additional parameters that can be taken to be common to all subjects, as discussed earlier.

In other words, despite considerable inter-subject differences in median latency, the model predicts the entire distributions generated by individual subjects rather accurately over the range of support values, both for correct and error responses, using very few parameters (two per participant plus two others).

## Discussion

It is generally agreed that reaction times reflect the time it takes for a decision signal to be accumulated to reach threshold, the noisiness of this process generating the random variability. One specific variety of this kind of model - random-walk, sometimes also called diffusion-drift - has been well understood for many decades^17^,^18^,^19^,^20^,^21^, and it is easy to show that statistically there is no more efficient way of detecting a sensory signal in the presence of background temporal noise^22^. Encouraged by the widespread use of RDK stimuli, whose detection must almost inevitably depend on some such process, this way of thinking has been increasingly adopted, indeed often now regarded as the *only* possible way of modelling RT data. It has come almost to be assumed that a mechanism essentially to do with detecting noisy stimuli ought to be able to explain all RTs, even when stimuli are *not* noisy.

But decision involves more than simply the detection of sensory evidence. In identifying visual objects there must be a logical distinction between *detection* - establishing the existence of individual features of the stimulus - and making a *decision* as to whether the resultant set of fragments of evidence enables one to deduce the existence of a particular object. To recognize that a letter is an E and not an F immediately suggests the idea of a hierarchy (though there are other possibilities): *detection* of individual lines and edges, followed by a *decision* about whether this particular collection of features favours the hypothesis that it is an F more than it does that it is an E^16^. Reddi^23^ has drawn a telling analogy with a court of law, where a clear distinction is made between evaluating individual items of evidence (gathered by a process of detection!) and making a decision as to whether, collectively, these fragments of evidence amount to a conviction.

These processes are necessarily logically distinct, working in contrasting ways. Detection is usually a matter of distinguishing a signal from background noise, for which random walk integration over time is the optimal procedure; decision introduces such Bayesian factors as prior probability and criterion level, as well as utility and reward. For the RDK, for instance, one can postulate an ensemble of units detecting local motion, followed by a second stage that uses this information to compute global motion (see for example)^24^. More specifically, one can postulate that each local detector raises a ‘flag’ to signal that they have seen their preferred stimulus; and then a subsequent and logically distinct mechanism that polls these flags to decide the overall motion (Fig. 7). We then have *two* integrator mechanisms in series: one detecting stimulus fragments, for which random walk is the optimum procedure (it need not be linear), and a second whose function is to collect information from a number of such fragments, and decide what is the best overall interpretation^25^. It is this second decision mechanism that constitutes the LATER model; unlike the first stage, it must make use of prior probabilities and is therefore likely to be essentially Bayesian in nature. The defining linearity of its accumulation is simply a consequence of the fixed signals generated by the raising of the ‘flags’ by the detectors forming the first stage. Random-walk and LATERian components both act as temporal integrators, but here behave differently because of the different inputs they receive: in the first stage, noise-dominated; in the second, noise-free, apart from the trial-to-trial randomness that is the defining feature of LATER. Either or both integrators can be leaky, provided the time-constant is long enough that its effects are not apparent over durations comparable with decision times.

Support for these general ideas – of two stages of decision, and of the internal generation of variability - has come from behavioural experiments^11^,^26^ and also from electrophysiology. We can for instance look at RTs to single low-contrast targets whose signal-to-noise ratio is low^16^,^27^. As this ratio is reduced, there is a transition from LATER-like distributions to those characteristic of random walk; as the external noise increases, so does the contribution of detection rather than decision to overall response time. Similarly, Schall and his colleagues have demonstrated a group of neurons in primate frontal eye fields that in certain kinds of discriminative task act in effect as stimulus detectors, and in turn drive movement-related neurons whose activity profile is a linear rise-to-threshold, the rate varying randomly on different trials^6^,^28^,^29^; and similar results have been obtained more recently from prefrontal cortex in a context-selective RDK task^30^. In addition, neurons in MT behave very like the ‘flag-raising’ postulated in our model, with brief periods of motion evoking activity lasting 200 ms or more^31^, implying the existence of a first stage of local integration; similar measurements of neuronal responses in monkey MT to very brief stimuli have been presented by Ghose and Harrison^32^. But suggestive as all this has been in supporting the idea of separate sequential stages of detection and decision, one could still object that even localized, ‘static’ stimuli might introduce sensory noise in the earlier stages of visual processing, or alternatively that patterns of activity observed in frontal cortex are not necessarily determinants of actual behaviour. The logical link between electrical activity of individual neurons in particular areas of the brain, and observed behaviour of the entire system, must in fact always be somewhat problematic, and cannot be regarded as providing knock-down evidence for the functional existence of two stages.

That internal noise might be an important determinant of variability in saccadic latency has quite often been suggested – for instance in the very explicit article by Reddi^23^, or in an earlier article that examined the broader functional consequences^33^. A subsequent paper^16^ went further in characterizing the two stages, and identifying the gradual transition from random walk to LATERian behaviour as sensory noise is reduced; but while this earlier work was suggestive, it was not conclusive. We felt therefore that it was essential to use a distributed stimulus, that necessarily demands a separate stage of decision performing spatial integration, so that we could not be accused of once again limiting ourselves to single, localized targets (for which it is not obvious that a decision as such is needed at all); and also to devise a way of eliminating as far as possible any contribution from a first, detection, stage, so that the action of the second stage would be revealed beyond dispute: for it is not the existence of the *first* stage that is in doubt, but that of the *second*. We do this by using a stimulus that because of its spatial extension necessarily demands a separate stage of decision that implements spatial integration, yet at the same time completely eliminates temporal noise: a drawback with the classic RDK stimulus is that by continually injecting noise into the decision system, it obscures the stochastic nature of the system itself, and to an extent the same objection may be made of experiments using masking^34^. Having eliminated these extraneous sources of noise, we find that the responses are still extremely variable and that their stochastic properties conform to LATER.

When a model successfully predicts observed data, it is not unreasonable to be asked to compare how well it does so in comparison with other models. In some circumstances (when the number of degrees of freedom are identical, and the model to be compared has a clear canonical form) this is perfectly appropriate. But for the principal models that might be considered rivals of LATER, these conditions are not met. Some, such as E-LATER^35^, or Brown’s ‘Linear ballistic accumulator^36^’ are derived from LATER, but with an added free parameter; consequently the model must *necessarily* generate a better fit to experimental data than LATER itself: this inevitable result tells us nothing about their relative merits. Important and popular alternative candidate models are those based on classic random walk (‘diffusion’) models, referred to earlier. A problem here is that random walk models exist in several different forms, and also tend to have quite large numbers of free parameters: for instance, no less than 15 in the Smith and Ratcliff two-stage model^26^, despite the use of smoothing and averaging across subjects. We are able to model entire distributions from individual subjects using very few parameters indeed: more specifically, our corpus of observations comprises over 200 DF, at a conservative estimate, yet the entire data set, comprising the individual distributions of each of the five subjects for each value of support, can be described by just two free parameters per participant (the slope and offset of the fitted linear relation between μ and support) plus two others common to all of them (σ and the fixed delay). It is perfectly possible that a diffusion model could perform equally well: but even leaving aside the number of free parameters, modelling one’s own data with one’s own interpretation of someone else’s model is actually unfair on the rival model, since one is not in a position to present it in its best light. If another author thinks their model can explain new data better, it is up to them to show that this is the case.

Another perfectly natural suggestion that at first sight seems very plausible is to attempt an explicit experimental comparison of RDT and RDK. Unfortunately this is not quite as straight-forward as one might at first suppose. Introducing prolonged temporal noise that can be integrated over time, entirely new considerations arise that have no equivalent in the RDT, in particular the well-known phenomenon of speed/accuracy trade-off. Stimuli such as the RDK allow one to make a quick decision using just the initial information, or – most obviously by raising the final threshold – to take longer and make a less uncertain choice. Both these features are evident in the results from the supplementary experiment shown in Fig. 3D. Here the duration of the RDK was 200 ms, which should in effect preclude the subject ‘thinking’ about the response during the trial. It can be seen that the very shortest latencies are similar to those of the RDT, the subject presumably responding in some trials to the start of the stimulus movement: but the longest latencies are increased by more than 100 ms. In addition, the overwhelming influence of the noise introduced by the RDK stimulus can be seen in the shallower slope: more than two-thirds of the variance of the RDK latency distribution is due to this extrinsic source of variability. In addition, Fig. 3E demonstrates that although the stimulus introduces extra noise, the extra integration time means that the responses are more accurate than for the RDT (84.5% as opposed to 69.8%). All of this is more-or-less what would be expected on general grounds; it does not shed much light on the question of two stages, partly because it cannot tell us whether these differences are due to slower detection by the first stage, or an alteration in the second stage by, in effect, an act of will acting on the criterion level^37^. A much clearer conclusion comes about, as we have argued above, not by adding more noise, but by forcing the problematic second stage to expose itself by as far as possible eliminating the external noise altogether.

Our finding that μ is a linear function of the support is equivalent to saying that it is proportional to the quantity of evidence received in favour of the corresponding hypothesis, as would be expected of a Bayesian mechanism. One dot detected as moving to the right provides evidence E_R_ for the hypothesis H_R_ that the overall motion is to the right, increasing the log likelihood by log *p*(E_R_|H_R_); it follows that the total increase will be proportional to the number of dots detected as moving in that direction; and the same applies for leftward motion. (This is similar, but not quite identical, to an argument presented in a previous paper^11^; the reason for the difference is that in that experiment the RDK stimulus had some dots moving randomly to left and right, and some coherently. This was also true of another study^38^, in which mean RTs – distributions were not analyzed – were found to be proportional to stimulus coherence, though the data were also compatible with a power-law relationship).

For simplicity we have regarded the two LATER units in Fig. 6 as calculating log probabilities, but there are other possibilities, for instance a pair of units that compute log odds rather than log probabilities: log[*p*(H_R)_/*p*(H_L_)]and log[*p*(H_L)_/*p*(H_R_)]. There is compelling evidence from monkeys making decisions on the basis of a series of partial items of information that at least some cortical neurons do indeed code for log odds^39^. Such a pair of units would receive excitatory connections from detection units for one direction, and inhibitory inputs from detection units for the opposite direction (there are attractive theoretical arguments for the utility of pairs of units coding for antithetical hypotheses in this way)^40^: this would be entirely compatible with our finding of a linear relation between support and μ, as would different weights being associated with different detection units, for instance from different parts of the visual field.

In summary, by using spatially extended stimuli and eliminating variability in the detection stage, what remains can only be assigned to a subsequent process, and not dismissed as something unique to the processing of localized targets, possibly by frontal rather than parieto-temporal mechanisms. Since it also provides clear evidence concerning the precise way in which these two stages are linked (by spatial summation of binary signals representing units of log likelihood), and thus by implication explains the peculiarity of LATER’s *linear* rise-to-threshold, and does all this by means of a quantitative model with a remarkably small number of free parameters, there is a sense in which the three components reinforce one another to produce a conclusion stronger than the sum of its parts. The introduction of a new type of stimulus, in many ways preferable to the ubiquitous RDK and likely to be widely adopted because of its obvious advantages, is an added bonus.

## Methods

### Participants

Five volunteers aged 20–21 participated in these experiments; none had visual defects other than deuteranopia in the case of participant A, and refractive errors, corrected as necessary; all but A and B (authors) were naïve as to the nature of the experiments. All participants gave informed consent, and the procedures used had been approved by a local ethics committee (the University of Cambridge Human Biology Research Ethics Committee) and were in accord with the Declaration of Helsinki.

### Eye movement recordings

We recorded eye movements using a dual differential infra-red reflection binocular oculometer placed on the bridge of the nose, determining eye position by comparing the reflectance from the sclera and the pupil of each eye, with 250 Hz bandwidth, linear to 7% within a range of ±30°^41^. A chin-rest minimized head movement. The oculometer’s output was sent to a ViSaGe system (Cambridge Research Systems Ltd, Kent, UK) sampling at 100 Hz in exact synchrony with the screen frame rate, and guaranteeing immunity against Windows-induced delays. The ViSaGe in turn interfaced with the recording and stimulation application SPIC^42^, that also generated the visual stimuli. Saccades were detected in real time using a criterion based on velocity and acceleration (normally 50 deg s^−1^ and 2500 deg s^−2^). After each run, we checked the individual saccadic traces manually; those containing obvious artefacts (due to blinks, head-movements or lapses of attention) or occurring with a latency of less than 60 ms were excluded from further analysis; they typically amounted to some 2% of the entire data set. The total numbers of saccades analyzed from each participant were: A, 1081; B, 1408; C, 743; D, 849; E, 1111.

### Visual stimuli

Participants sat 1 m away from the CRT screen displaying the visual stimuli (GDM-F520 monitor, resolution 800 × 600; Sony, Tokyo, Japan), subtending roughly 22° × 17° of visual angle. We arranged the ambient lighting to be of similar luminance to the screen, to reduce adaptational effects. The frame rate of the monitor was 100 Hz, with interlacing, and a phosphor persistence of some 5 ms, factors that limit the uncertainty of timing of any particular element to not more than 10 ms.

**Figure 1:**
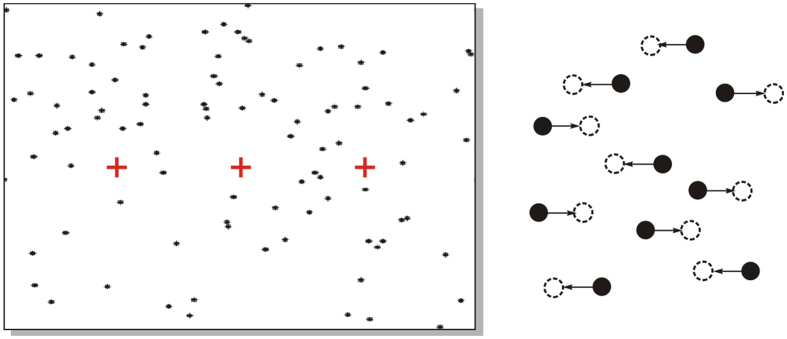
The stimulus. Left, a field of random dots, with crosses forming the central fixation point and peripheral targets. Right, enlarged view of part of the screen: at time zero, a specified proportion of the dots move a fixed distance just once horizontally (dotted circles) to the left and the others the same distance to the right. In this *Random Dot Tachistogram* (RDT) the movement is essentially instantaneous, and after it occurs the appearance of the screen conveys no information relevant to the task. For more details, see *Methods*.

**Figure 2:**
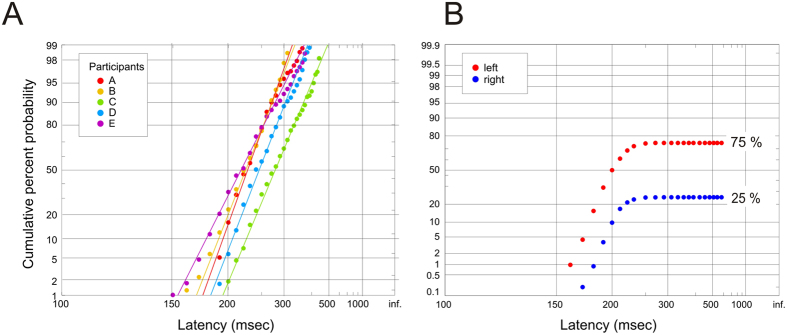
Reciprobit plots. (**A**) Cumulative histograms for each subject of reaction time distributions for 100%, 98% and 95% support for a particular direction, shown as ‘complete’ reciprobit plots, i.e. as a proportion of *responses in that direction only*. The lines are the best-fit expectations from the LATER model. In each case the data are very well fitted by the model, as shown by the superimposed lines, which are obtained by best-fit of μ and σ by minimization of the Kolmogorov-Smirnov one-sample test: *p* = 0.87, 0.93, 0.94, 0.85, 0.85. (**B**) The expected form of the reciprobit plots for responses to left and right to a stimulus in which the leftward support is high and the rightward support correspondingly low. They are plotted as ‘incomplete’ distributions, i.e. as a proportion of the *total responses in both directions* (whereas the complete distributions in A represent the proportion of responses in the same direction only). Consequently they level off to give the final response rates in each direction, in this case 25% to the right and 75% to the left.

The saccadic targets were two red (CIE *x* = 0.624, *y* = 0.341, Lum = 22.3 cd m^−2^) crosses spanning 1° of visual angle, on a white (CIE 0.276, 0.300, 118) background, 10° to the left and to the right of an identical red fixation cross at the centre of the screen (Fig. 1, left). In addition, there was a field in which 100 black dots (CIE 0, 0, 0, diameter 0.2°) were randomly-positioned, covering the entire extent of the screen; since dots sometimes land on each other (though this was minimized by using the Sobol method for two-dimensional random number generation), the actual number of dots displayed will sometimes have been less than 100.

### Protocol

A single trial consisted of three parts: an inter-trial period of 550 ms during which the participant refoveated the central target, a random ageing^43^ fore-period lasting between 200 and 900 ms during which the stationary field of dots was presented, terminating in a once-and-for-all 1° displacement of the dots, a predetermined proportion to the left and the remainder to the right (Fig. 1, right); the dots remained stationary in their new positions until the end of the trial. Subjects were required to indicate the perceived overall direction of movement by making a saccade to one of a pair of red target crosses on each side of the central fixation cross, and in the final recording period the reaction time (RT) of this response was measured. SPIC terminated each trial 50 ms after completion of a saccade, or 5000 ms after the beginning of the trial, whichever was shorter.

A larger proportion of dots moving in a particular direction affords greater support to the hypothesis that this is the overall majority direction. For example, if 85% in a particular trial move to the left, and 15% to the right, and the subject responds with a leftward response, we can say that the stimulus provided 85% support to this decision; had the saccade been to the right, the degree of support for that response would have been 15%. A single run consisted of 200 randomly-interleaved trials, using the following set of values of support to the right: 100, 98, 95, 85, 75, 65, 55, 45, 35, 25, 15, 5, 2, 0%. A complete data set contained multiple runs with a rest between each run. We allowed subjects some practice at the start, to get accustomed to the task, the data being discarded.

**Figure 3:**
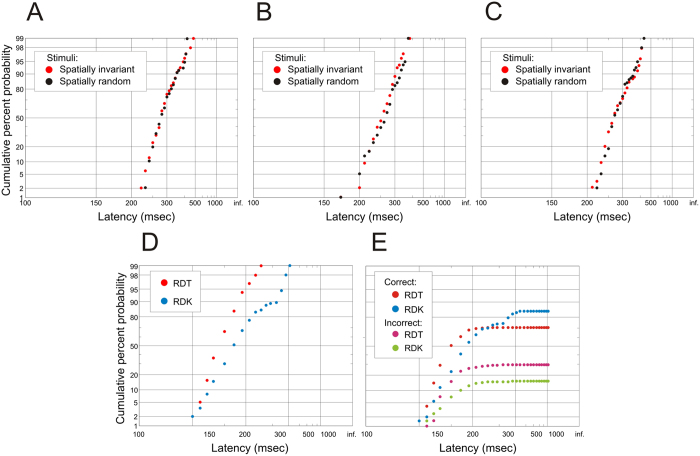
Sources of external noise. (**A**–**C**) Reciprobit plots of latency distributions in three subjects for responses to 25%:75% RDT targets which were either randomly different on each trial, both in initial configuration and in subsequent movement (•), or identical in both respects (•). In no case are the distributions significantly different (KS 2, *p* > 0.1). (**D**,**E**) Reciprobit plots of latency distributions in one subject for an RDT stimulus and an RDK stimulus of duration 200 ms: the complete distributions in (**D**) illustrate the increased variance introduced by the RDK (the corresponding values of σ^2^ are 0.18 for RDT and 0.62 for RDK). The incomplete distributions in (**E**) show that the RDK responses are more accurate, since they permit integration over a longer period; the asymptotes for correct responses are 69.8% for RDT and 84.5% for RDK.

**Figure 4:**
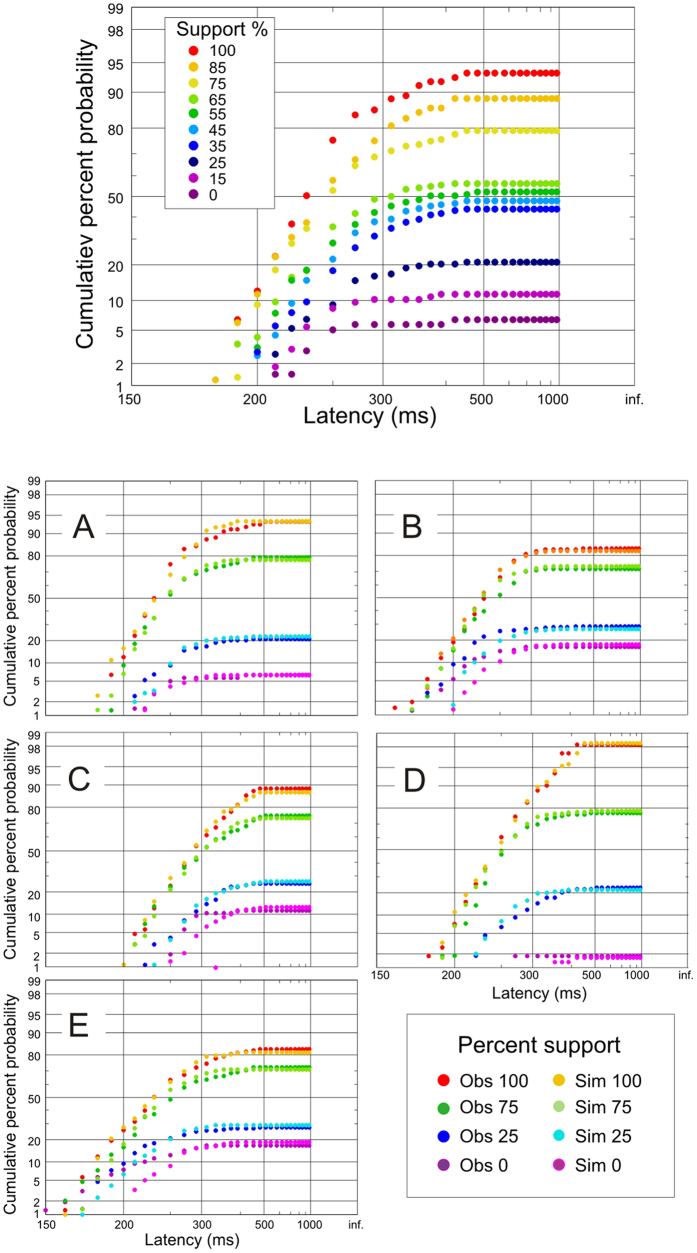
Observed and simulated reaction time distributions. Above: ‘incomplete’ reciprobit plots (as in Fig. 2B) for subject A for all of the different degrees of support. Below: comparison of observed (Obs) and simulated (Sim) distributions for the supports shown, for all participants.

**Figure 5:**
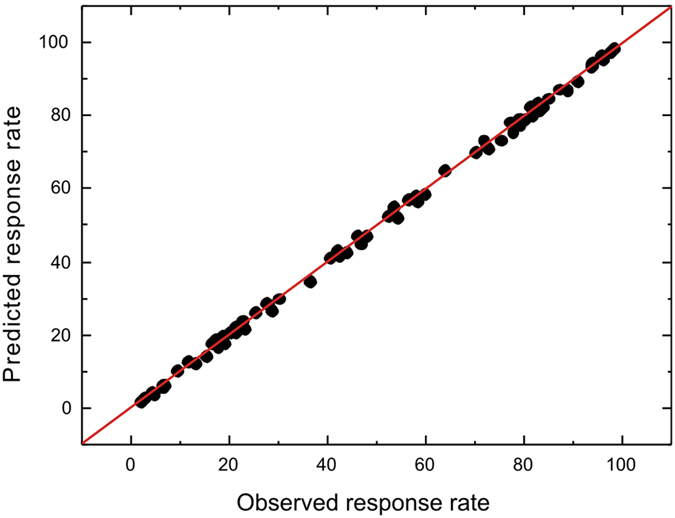
Accuracy of the model in predicting response rates. The points show the relationship between the observed and simulated final response rates for all participants and degrees of support. The line represents the exact equality between simulations and observations expected if the model’s predictions were perfect.

**Figure 6:**
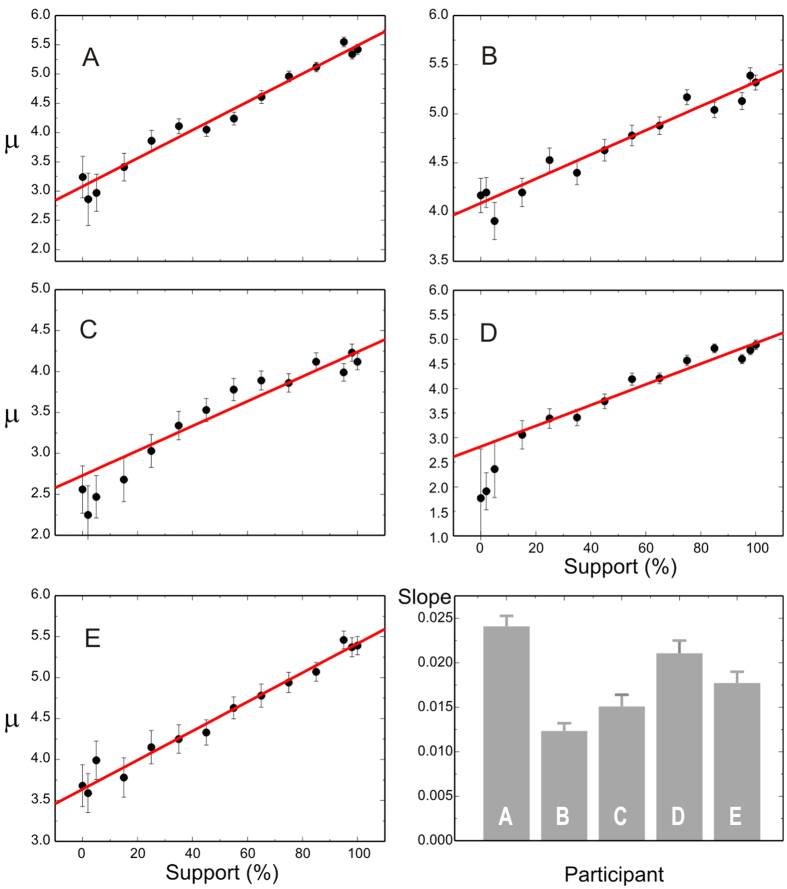
Relation between μ and degree of support. (**A**–**E**) Best-fit values of μ (Hz) as a function of support for all participants. The lines show the result of weighted linear regressions; the error bars show ±1 SE, necessarily larger for low support because the data-sets are smaller. Pearson’s correlation coefficients for each participant were as follows: (**A**) *R *= 0.982; B, 0.967; C, 0.942; D, 0.942; E, 0.989). Bottom right: the values of the slopes of these lines (Hz per percent, ±1 SE) for each participant.

**Figure 7:**
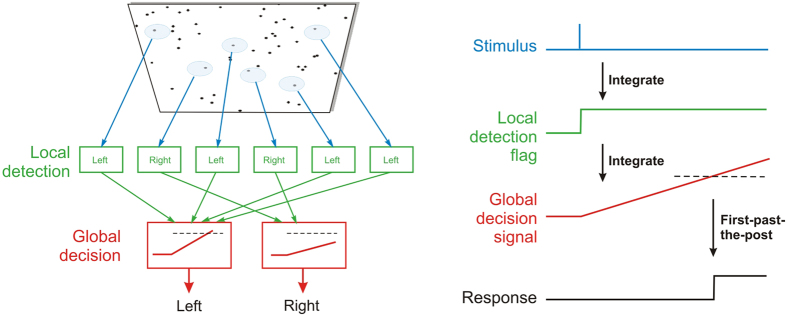
Model of detection and decision making in this task. Left, local information about motion direction (top) activates corresponding localized detection units (green) that in turn send their output to the appropriate LATER decision unit (red); the more input a LATER unit receives from the detectors, the greater the mean rate of rise of the decision signal and therefore the more likely it is to initiate a corresponding movement. Right, the underlying neural signals. The stimulus is transient, and is integrated by the detection process to raise a ‘flag’. The set of such flags must then be integrated again by the global decision units that race against each other: the winner triggers the response.

In a supplementary experiment undertaken by three of our subjects (Fig. 3A–C), we removed spatial as well as temporal randomness, by presenting the same starting pattern and dot movement on each trial for a particular degree of support (25:75%); in every other respect the protocol was as previously described. In a second supplementary experiment (Fig. 3D,E), designed to demonstrate directly the effect of the extra noise introduced by conventional RDK stimuli, in control runs we presented a single RDT stimulus in each trial, with a displacement of 0.1 deg and with 35:65% support (35% of the dots moving coherently, the rest with random direction), but otherwise as in the main experiments; in experimental runs, after the usual random foreperiod, RDK was displayed for 200 ms, with a 0.1 displacement every 20 ms (thus resulting in a total displacement of 1 deg for the coherently-moving population of dots), and the same support values of 35:65%.

### Data modeling

To model the behaviour in these experiments we used two parallel LATER units, corresponding to the two directions, that raced to a common constant unit threshold with different mean rates of rise, dependent on the degree of support for their respective directions, but with the same standard deviation σ. (We included a delay of 50 ms, representing the fixed delays corresponding to photoreceptor activation, conduction velocity, synaptic delay etc). One of these LATER units led to ‘correct’ saccades (with mean rate of rise μ_c_) and the other to ‘incorrect’ saccades (with mean rate of rise μ_i_): that is, saccades respectively in accordance with the overall direction of motion, and in the opposite direction. Since the ‘correct’ unit has higher support than the ‘incorrect’ one, the corresponding value of μ can be expected to be higher, generating a shorter RT and a greater likelihood of winning; but because of the trial-by-trial variability that is a feature of LATER, incorrect responses will inevitably be made, more frequently when their support is higher.

This model, implemented in SPIC, was used as the basis for a simulation to determine the best-fit values of the parameters to the observed distributions, by reiterative minimization of the Kolmogorov-Smirnov two-sample statistic in simulated runs of 300 trials each. Previous papers have described the general procedures^44^,^45^,^46^. The simulation fitting uses the Amoeba algorithm^47^, performing an adaptive exploration of the region round the previous best estimate, and reducing the size of the corresponding simplex each time until no further significant improvement occurs: this typically requires some 10–20 iterations. Preliminary results indicated that of the three parameters, μ_i_, μ_c_ and σ, while the first two varied substantially between subjects for a given degree of support, σ did not, and in fact had relatively little influence on the goodness of fit. To reduce the amount of computation and the number of unnecessary free parameters we therefore used a value of σ = 1.0 for all participants except E, for whom σ = 1.3 was used; allowing ourselves to specify σ for each individual would clearly have improved the quality of the fits still further, but it seemed already to be more than adequate. Thus the only two parameters estimated from the data for each subject were μ_i_ and μ_c_, the mean rates of rise for incorrect and correct responses, for each degree of support.

## Additional Information

**How to cite this article**: Genest, W. *et al*. The random dot tachistogram: a novel task that elucidates the functional architecture of decision. *Sci. Rep.* **6**, 30787; doi: 10.1038/srep30787 (2016).
